# Prevalence and correlates of tobacco use in Botswana: evidence from the 2014 Botswana STEPwise survey

**DOI:** 10.1186/s12889-022-14879-y

**Published:** 2023-01-06

**Authors:** Mpho Keetile, Kagiso Ndlovu, Naomi Setshegetso, Sanni Yaya, Fattimah Serojane

**Affiliations:** 1 Department of Population Studies, University of Botswana, Gaborone, Botswana; 2 Department of Computer Science, University of Botswana, Gaborone, Botswana; 3 Department of Economics, University of Botswana, Gaborone, Botswana; 4 School of International Development and Global Studies, University of Ottawa, Ottawa, Canada; 5 Public Health Department, Institute of Health Sciences, Lobatse, Botswana

**Keywords:** Prevalence, Correlates, Current tobacco use, Botswana

## Abstract

**Background:**

Tobacco use is one of the notable risk factors for non-communicable diseases globally. The objective of this study was to assess the prevalence of tobacco use and identify its correlates in the general population of Botswana aged 15 to 69 years.

**Methods:**

This study used a nationally representative WHO STEPwise Approach to Non-Communicable Disease Risk Factor Surveillance (STEPS) risk factors conducted in 2014 to explore the prevalence of tobacco use and its correlates in Botswana. Using IBM SPSS version 27, data on 4062 people aged 15 to 69 years who had been selected using multistage cluster sampling and had successfully completed the individual questionnaire were analysed. The prevalence of current tobacco smoking and smokeless tobacco use was determined using descriptive statistics while multivariable logistic regression was employed to assess correlates of current tobacco smoking and smokeless tobacco use. All comparisons were statistically significant at 5% significance level.

**Results:**

From a total sample of 4062 participants the prevalence of current tobacco smoking was estimated to be 12.9% while smokeless tobacco use was 3.2%. Adjusted results indicate that the odds of current tobacco smoking were eight times (AOR = 8.57, C.I = 6.28-11.7) higher among males compared to their female counterparts; six(AOR = 6.52, C.I 3.64-11.6) and three (AOR = 3.27, C.I. =2.07-5.15) times higher among respondents with no education and primary level education respectively, compared to their counterparts with tertiary or higher education; while for alcohol users the odds of current tobacco smoking were four times (AOR = 4.28, C.I = 2.93-6.24) higher than among non-alcohol users. The odds of smokeless tobacco use were significantly higher among women compared to men (AOR = 7.34, C.I = 4.01-13.4); individuals aged 50-59 (AOR = 1.15, C.I = 1.06-3.37) and 60-69 years (AOR = 1.23, C.I. =1.08-3.63) compared to 15-29 years; individuals with no education (AOR = 2.07, C.I = 1.03-4.02) and primary education (AOR = 1.05, C.I = 1.01-2.23) compared to individuals with tertiary education. However, the odds of smokeless tobacco use were significantly lower among individuals who consume alcohol (AOR = 0.48, C.I. = 0.29-0.80) compared to non-alcohol consumers.

**Conclusion:**

Findings of this study indicate the need to strengthen existing national policies to reduce harmful use of tobacco among men, women, older adults, no or primary education level individuals and alcohol users.

## Background

Globally, the use of tobacco has been noted as one of the leading risk factors for non-communicable diseases (NCDs) [1]. As a result, tobacco use is a major public health concern and a modifiable cause of morbidity and mortality. According to the World Health Organization (WHO) report over 1.1 billion people smoked tobacco globally in 2015, which represents around 15% of the global population [2]. On the other hand, tobacco use is predicted to kill around 6 to 7 million people annually [1, 3, 4]. Most (80%) of these individuals who have been killed by tobacco live in low- and middle-income countries (LMICs) [3, 4]. Moreover, tobacco smoking has been attributed to 150 million disability-adjusted life years and 11.5% of total global deaths [3].

It is estimated that in Sub Saharan Africa (SSA), around one in five adults smoke tobacco products [5]. This makes SSA to have low prevalence of tobacco use compared to other regions of the world [6, 7]. Most of the countries in SSA are in the first stage of the tobacco epidemic continuum which is a notion that nicotine and tobacco products fall along a spectrum from most to least risk, which denotes the impact of tobacco use on human health at the individual and population level [7]. In this stage there is higher prevalence of smoking among men compared to women [7]. A recent systematic review by Brathwaite et al. [7] conducted using studies published between 2007 and 2014 found that men smoked tobacco products more than women in all the SSA countries represented. More recently a study using the latest Global Adult Tobacco Survey (GATS) data by Chandrashekhar et al. [8] found that current tobacco smoking varied widely between countries among men and women. For instance, the prevalence of current tobacco use among male participants ranged from 4.5% in Ghana to 46.0% in Lesotho during the most recent surveys. Except for Lesotho, which also had the highest rate of tobacco use, in all other SSA countries except Zambia, tobacco use rates had significantly decreased in terms of prevalence rates ranging from 1.5% (Ghana) to 9.6% (Sierra Leone) [8]. The highest percentage decrease of tobacco smoking (43.7%) was in Cameroon (from 14.5 to 8.1%) between 2011 and 2018 [8].

Botswana has fully adopted the WHO best buys strategies for tobacco use. However, it is among the countries with high smoking prevalence in the Southern Africa region, especially among men [9]. The 2008 WHO STEPwise survey results showed that prevalence of current smoking was 19.7% among people aged 25-64 years and the average age of initiation of daily smoking for the general population was 23.6 years [9]. The latest GATS data indicate that current tobacco use among people aged 15+ years in Botswana was estimated to be 17.6% for both men and women in 2017 [10]. Despite the high prevalence of tobacco use, there is paucity of information about factors influencing tobacco use in Botswana. Consequently, little is known about correlates of tobacco use among people aged 15-69 years. The main objective of this study was to assess prevalence and correlates of tobacco use in the general population of Botswana. Since this study is the first comprehensive national analysis of the correlates of tobacco use in the general population, it provides evidence for a more targeted programmatic response to tobacco use in the country.

## Materials and methods

### Study design

The current analysis uses data from a cross-sectional, population-based survey called the Botswana WHO STEPwise Survey II which was conducted in 2014 based on people aged 15-69 years. STEPS Survey II used a multi-stage cluster sampling methodology to arrive at nationally representative sample. A detailed report on the methodology of the Botswana STEPS Survey has been presented elsewhere [11]. This study is based on secondary data analysis of the population-based 2014 Botswana STEPS survey.

### Sampling procedure

A multi-stage stratified sampling procedure was used in the 2014 Botswana STEPS Survey; therefore, the use of standard statistical methods for analyzing the data would produce unreliable estimates of the desired parameters [5]. As a result, during analysis of data, the complex sample module in SPSS was used to cater for the multiple stages of sampling. The target sample size for the 2014 Botswana STEPS Survey was 6410 but 4074 individuals participated, yielding an overall response rate of 64% [11]. From a total sample of 4074 individuals who completed the STEPS survey questionnaire, only 4062 successfully completed and responded to the tobacco use section of the questionnaire.

### Measures

#### Dependent variables

The main outcomes of interest for the study are current tobacco smoking and current smokeless tobacco use. Current tobacco smoking was derived from the question; ‘Do you currently smoke any tobacco products, such as cigarettes, cigars or pipes?’. Current tobacco smoking involves the combustion of the tobacco product and inhalation of tobacco smoke through the mouth [8]. Current smokeless tobacco use was derived from the question asking respondents; ‘Do you currently use any smokeless tobacco products such as snuff, chewing tobacco, betel?’ Smokeless tobacco products are those whose use involves chewing the tobacco product or sniffing it through the mouth or nose [8]. Current tobacco smoking and current smokeless tobacco use were coded into binary variables, with codes ‘yes = 1’ indicating tobacco use and ‘no = 0’ indicating non-use of tobacco for both current tobacco smoking and smokeless tobacco use.

#### Independent variables

The selection of the independent variables used for analysis was based on literature review [12] and on the availability of the limited socio-demographic variables collected during the 2014 Botswana STEPS survey. The following sociodemographic variables were used as covariates of tobacco use: sex, age, marital status, education level, and employment status. Categories for age were: 15-29, 30-39, 40-49, 50-59 and 60-69 years. Marital status was coded as currently married, formerly married (widowed, divorced and separated) and never married (includes cohabiting). Education was categorized into, no education, primary, secondary and tertiary or higher education level. Employment status was created based on the participants’ main work status over the past year; public sector (government employee, parastatal), private sector (non-governmental employee), self-employed, unpaid/students (non-paid/unpaid family helper, student), homemaker/house worker, and unemployed (able to work and unable to work, retired). Alcohol consumption was also used as an independent variable consistent with available literature that has shown that tobacco use is associated with alcohol consumption [13]. Alcohol consumption was derived from a question asking respondents whether they have consumed alcohol in the past 12 months before the survey. Information about the dangers of smoking was also used as an explanatory variable consistent with literature [14]. This variable was derived from a question which asked respondents whether they received any information about the dangers of smoking in the past 30 days.

### Statistical analyses

In the first level of analysis descriptive statistics, frequency and proportions are presented. A chi-square (χ^2^) statistic was used in bivariate analyses to assess the association between current tobacco smoking and smokeless tobacco use and sociodemographic and behavioral variables. Weights were created within the dataset and the weights command was activated during analysis to produce weighted estimates for both bivariate and multivariate analyses.

In multivariable analyses we included all independent variables that were conceptually associated with tobacco use [15]. All independent variables were used as covariates of current tobacco smoking and current smokeless tobacco use. The variables, alcohol consumption and information about the dangers of tobacco use were hypothesized to be important factors given their known association with sociodemographic factors and tobacco use [16]. In the tables for results we present unadjusted (OR) and adjusted odds ratios (AOR) for variables included in the models. Both unadjusted and adjusted odds ratios are presented with associated 95% confidence intervals (CIs). All the statistical analyses were done using IBM SPSS version 27.

### Ethical considerations

This study uses secondary data provided by the Ministry of Health and Wellness hence no ethical protocols were needed for this study. However, during the survey, the survey’s protocol was reviewed and approved by the Ministry of Health and Wellness Institutional Review Board. Informed Consent was obtained from participants and parents/LAR (legally authorized representative) in case of minors and no individually identifiable information was collected [9].

## Results

### Characteristics of the study population

There were 4062 participants sampled for analysis in this study and 12.9% of them were currently smoking tobacco while 3.2% were currently using smokeless tobacco (Table 1). Using a chi-square (χ^2^) statistic and weighted percentages, a significantly higher proportion among males (28.6%), individuals with no-education (43.5%) and people in ages 50-69 years (13.9%), self-employed individuals (20.3%), never married (12.0%), and alcohol consumers (31.3%) reported current tobacco smoking. Current smokeless tobacco use was significantly higher among; females (4.0%), people aged 60-69 years (10.2%), individuals with no education (20.8%), formerly married individuals (8.7%), and home makers (4.9%).

**Table 1:** Breakdown of tobacco use variables by sociodemographic characteristics of the population, Botswana WHO STEPwise Survey II-2014

Characteristic	Current tobacco smoking		Current smokeless tobacco use		***Total (N)***
	***Yes(n)***	***Weighted %***	***Yes(n)***	***Weighted %***	
**Sex**					
Male	376	28.6^a^	20	1.5^a^	1316
Female	74	2.7	110	4.0	2746
**Age groups**					
15-29	174	11.1^a^	17	1.1^a^	1564
30-39	109	11.0	36	3.2	987
40-49	94	13.9	30	4.3	674
50-59	19	13.9	61	9.1	674
60-69	19	6.8	28	10.2	276
**Education level**					
No education	166	43.5^a^	79	20.8^a^	382
Primary	136	14.1	92	9.6	963
secondary	210	10.9	44	2.3	1925
Tertiary or higher	79	10.0	5	0.6	792
**Marital status**					
Currently married	128	10.8^a^	52	4.4^a^	1186
Formerly married	19	8.0	21	8.7	240
Never married	316	12.0	58	2.2	2636
**Employment status**					
Public sector	11.2	63^a^	11	2.0^a^	565
Private sector	16.5	88	11	2.0	536
Self-employed	18.2	89	21	4.4	487
Unpaid/students	9.1	61	14	2.1	670
Home maker/house worker	11.3	42	18	4.9	370
Unemployed	12.7	182	57	4.0	1430
**Alcohol consumption in the past 12 months**					
Yes	349	31.3^a^	53	4.8^a^	1115
No	24	4.9	10	2.1	493
**Information about the dangers of smoking in the past 30 days**					
Yes	378	11.6	1224	3.2	3989
No	43	10.3	2	0.3	73
Total		12.9		3.2	4062

^a^Statistically significant at 5% significance level. A chi-square (χ2) statistic was used in bivariate analyses to assess the association between current tobacco smoking and smokeless tobacco use and sociodemographic and behavioral variables

### Correlates of current tobacco smoking

The adjusted logistic regression model results indicates that the odds of current tobacco smoking were more than eight times (AOR = 8.57, C.I. = 6.28-11.70) higher among males compared to their female counterparts (Table 2). On the other hand, the odds of current tobacco smoking were significantly low among individuals in ages 60-69 (AOR = 0.32, C.I. = 0.16-0.64) compared to people in ages 15-29 years, while for other age groups there were no variations in current tobacco smoking compared to individual aged 15-29 years. On the other hand, the odds of current tobacco smoking were six (AOR = 6.52, C.I 3.64-11.6) and three (AOR = 3.27, C.I. =2.07-5.15) times higher among individuals with no education and primary education level, respectively, compared to their counterparts with tertiary or higher education. The odds of current tobacco smoking were four times (AOR = 4.28, C.I. = 2.93-6.24) higher among people who consumed alcohol in the past 12 months compared to those who did not consume alcohol in the past 12 months. There was no significant variation between current tobacco smoking based on the marital status, employment status and access to information about the dangers of smoking in the past 30 days.

**Table 2:** Covariates associated with current tobacco smoking in Botswana, Botswana WHO STEPwise Survey II-2014

Current tobacco use	Crude odds ratios OR (95% CI)	***p***-value	Adjusted odds ratio AOR (95% CI)	***p***-value
**Sex**				
Male	0.09 (0.07-0.11)	0.00	8.57 (6.28-11.7)	0.00
Female	1.00		1.00	
**Age groups**				
15-29	1.00		1.00	
30-39	1.00 (0.77-1.30)	0.97	0.98 (0.69-1.40)	0.90
40-49	1.02 (0.76-1.37)	0.86	1.16 (0.74-1.81)	0.51
50-59	0.93 (0.68-1.26)	0.64	0.80 (0.46-1.38)	0.42
60-69	0.61 (0.12-1.12	0.36	0.32 (0.16-0.64)	0.01
**Education level**				
No education	0.33 (0.24-0.46)	0.00	6.52 (3.64-11.6)	0.00
Primary	0.65 (0.49-0.88)	0.00	3.27 (2.07-5.15)	0.00
secondary	0.89 (0.67-1.17)	0.40	1.36 (0.94-1.96)	0.09
Tertiary or higher	1.00		1.00	
**Marital status**				
Currently married	1.20 (0.97-1.48)	0.08	0.87 (0.63-1.21)	0.41
Formerly married	1.18 (0.78-1.78)	0.42	1.22 (0.60-2.48)	0.57
Never married	1.00		1.00	
**Employment status**				
Public sector	1.16 (0.85-1.57)	0.33	0.75 (0.48-1.15)	0.19
Private sector	0.73 (0.56-0.97)	0.03	0.78 (0.52-1.17)	0.23
Self-employed	0.65 (0.49-0.86)	0.03	0.83 (0.55-1.26)	0.40
Unpaid/students	1.45 (1.07-1.97)	0.01	0.76 (0.48-1.21)	0.25
Home maker/house worker	1.14 (0.80-1.63)	0.46	1.65 (0.97-2.82)	0.06
Unemployed	1.00		1.00	
**Alcohol consumption in the past 12 months**				
Yes	0.18 (0.13-0.26	0.00	4.28 (2.93-6.24)	0.00
No	1.00		1.00	
**Information about the dangers of smoking in the past 30 days**				
Yes	1.00		1.00	
No	0.56 (0.31-1.02)	0.06	1.67 (0.66-4.24)	0.27

All comparisons statistically significant at 5% level, adjusted for sex, age, education level, marital and employment status

*OR* Crude Odd Ratios, *AOR* Adjusted Odd Ratios, *CI* Confidence Interval

### Correlates of current smokeless tobacco use

In the adjusted analyses, the odds of smokeless tobacco use were observed to be seven times (AOR = 7.34, C.I. = 4.01-13.4) higher among females compared to their male counterparts (Table 3). For age, the odds of smokeless tobacco use were significantly high among individuals aged 50-59 (AOR = 1.15, C.I. = 1.06-3.37) and 60-69 years (AOR = 1.23, C.I. =1.08-3.63) than among 15–29-year-olds. For education, the odds of smokeless tobacco use were significantly higher among individuals with no education (AOR = 2.07, C.I. = 1.03-4.02) and primary education (AOR = 1.05, C.I. = 1.01-2.23) compared to tertiary education. It was also observed that the odds of reporting smokeless tobacco use were significantly lower (AOR = 0.48, C.I. = 0.29-0.80) among individuals who consumed alcohol compared to non-alcohol consumers. There was no significant statistical association between marital status, employment status and having received information about the dangers of smoking in the past 30 days and reporting to have used smokeless tobacco. However, in the crude logistic regression model, these variables were significant but non-significant after introduction of control variables suggesting that their association with smokeless tobacco use was spurious.

**Table 3:** Covariates associated with current smokeless tobacco use in Botswana, Botswana WHO STEPwise survey-2014

Current smokeless tobacco use	Crude odds ratios OR (95% CI)	***p***-value	Adjusted odds ratio AOR (95% CI)	***p***-value
**Sex**				
Male	1.00		1.00	
Female	4.51 (2.88-7.04)		7.34 (4.01-13.4)	0.00
**Age groups**				
15-29	1.00		1.00	
30-39	0.43 (0.27-0.68)	0.00	0.40 (0.18-0.86)	0.19
40-49	0.39 (0.24-0.63)	0.00	0.33 (0.14-0.77)	0.11
50-59	0.17 (0.11-0.26)	0.00	1.15 (1.06-3.37)	0.00
60-69	0.13 (0.09-0.19)	0.01	1.23 (1.08-3.63)	0.00
**Education level**				
No education	3.02 (1.10-4.07)	0.00	2.07 (1.03-4.02)	0.00
Primary	2.04 (1.01-3.11)	0.00	1.05 (1.01-2.23)	0.00
secondary	1.20 (0.07-2.57)	0.27	1.16 (0.03-3.72)	0.13
Tertiary or higher	1.00		1.00	
**Marital status**				
Currently married	0.67 (0.50-0.91)	0.01	0.91 (0.55-1.51)	0.73
Formerly married	0.28 (0.18-0.43)	0.00	0.67 (0.31-1.45)	0.31
Never married	1.00		1.00	
**Employment status**				
Public sector	2.68 (1.59-4.51)	0.00	1.47 (0.68-3.15)	0.32
Private sector	2.86 (1.65-4.96)	0.00	1.04 (0.47-2.32)	0.90
Self-employed	2.18 (1.31-3.63)	0.00	1.35 (0.66-2.77)	0.40
Unpaid/students	1.77 (1.17-2.68)	0.00	1.22 (0.54-2.73)	0.62
Home maker/house worker	1.02 (0.66-1.57)	0.92	1.59 (0.75-3.38)	0.22
Unemployed	1.00		1.00	
**Alcohol consumption in the past 12 months**				
Yes	0.92 (0.59-1.43)	0.71	0.48 (0.29-0.80)	0.00
No	1.00		1.00	
**Information about the dangers of smoking in the past 30 days**				
Yes	1.00		1.00	
No	0.90 (0.32-2.51)	0.84	1.03 (0.21-5.00)	0.96

All comparisons statistically significant at 5% level, adjusted for sex, age, education level, marital and employment status

*OR* Crude Odd Ratios, *AOR* Adjusted Odd Ratios, *CI* Confidence Interval

## Discussion

This study provides the nationally representative estimates on the prevalence of tobacco use in Botswana. Overall, 12.9% of the respondents reported that they were currently smoking tobacco while 3.2% reported that they were using smokeless tobacco. The prevalence of both current tobacco smoking and smokeless tobacco use is comparatively low compared than in most sub-Saharan Africa countries. For example, countries such as South Africa (15%), Algeria (21.8%), Burkina Faso (19.8%) and Zambia (15.8%) have higher current tobacco use [8, 10]. The plausible explanation for the comparatively low prevalence rate of tobacco smoking in Botswana is the rigorous campaigns, including tobacco advertising ban together with other educational programmes aimed at different sectors of the population and the general public. The efforts may have contributed to sensitizing the general public about tobacco products’ harmful effects on human health and fostered a positive political climate [17].

Sex differences were observed in current tobacco smoking, with men eight times more likely to report current tobacco smoking compared to women. This finding is consistent with findings from other studies in SSA, which found that smoking prevalence rates tend to be consistently higher among men than women [18–20]. While biomedical literature suggests that the lower consumption of tobacco among women may be related to sex differences in motivations for smoking [20–22], psychological literature on the other hand posits that sex differences in tobacco smoking are mainly due to different behaviours, having its roots in traditional sex roles [23, 24]. The latter seems to be relevant to Botswana context where traditional sex roles lead to social pressure against female smoking [25]. Moreover, traditional sex norms cause differences in personal characteristics leading to acceptance of smoking among men than women [26].

Quite conversely after adjusting for cofounders the odds of smokeless tobacco use were found to be seven times higher among women than men. This finding contradicts most findings in LMICs [20–23]. The plausible explanation for the high prevalence of smokeless tobacco use among women can be attributed to the common anecdotal beliefs in Botswana that smokeless tobacco especially snuff is good for relieving stress, depression and hypertension. Smokeless tobacco use was significantly higher among individuals aged 50-69 years than among 15–29-year-olds and this could also be attributed to the anecdotal beliefs that it relieves stress, depression and hypertension which are more common among the older adults.

The odds of current tobacco use were found to be lower in ages 60-69 years while for other age groups there were no variations in current tobacco use, compared to 15-29 years. This finding is not consistent with other previous studies from countries such as Italy [27], Germany [28], Brazil [29], South Africa [30] and Zambia [31] which found that the odds of smoking were highest among older adults, than adolescents and middle-aged adults. Tobacco use among young adults in Botswana could be attributed to the fact that smoking is socially acceptable, and it is perceived to be glamorous to smoke even though much is known about the health risks of smoking. Cumulative effects of smoking during adolescence may predispose the young adult population to tobacco-caused diseases in later life [32].

For education, we found that people with no education or primary education were more likely to report current tobacco smoking compared to their counterparts with tertiary or higher education. Similarly, smokeless tobacco use was found to be significantly higher among individuals with no education or primary education. Pampel [26] using population-based data from 16 Demographic Health Surveys (DHS) found that tobacco use in SSA was more prevalent among low education populations. This suggests that higher education level seems to have a protective effect against tobacco smoking. This is because individuals with higher education are often exposed to disease prevention services and sufficient access to health promotion information compared to the less educated [33].

We found that people who reported alcohol use were four times more likely to smoke tobacco. Consistent with this study, many studies have found that alcohol consumption and tobacco smoking are closely related behaviours [17, 18, 34]. Thus, not only are the people who drink alcohol more likely to smoke (and vice versa) but also people who drink larger amounts of alcohol tend to smoke more tobacco products [35]. Contrarily the odds of smokeless tobacco use were significantly low among alcohol users. This connection has been observed in few cross-sectional studies that were also not precisely intended to reveal the likely sequence of substance use behaviours [36–40]. Drinkers have been found to be more likely to use smoked tobacco products than smokeless tobacco products [41]. Given the direction of the relationship between alcohol consumption and tobacco use, future studies should focus on the influence of alcohol use on other substances in order to come up with effective holistic interventions.

### Strengths and limitations

To the best of our knowledge, this is one of the few national cross-sectional studies on the prevalence, patterns and correlates of tobacco use in Botswana. Patterns of tobacco use show age, gender, educational differences in the sampled population. Furthermore, smoking was more prevalent among alcohol users than non-alcohol users. This cross-sectional study was not designed to infer causality and there is no data to assess the temporal sequence of tobacco use. There is also a possibility of under-reporting of tobacco use due to the cultural implications and potential stigmatisation. Moreover, although this is the latest survey with tobacco use among adults in Botswana tobacco use patterns have likely evolved since 2014 when the data was collected.

## Conclusion

This study provides vital insights into understanding prevalence of tobacco use (current smoking and smokeless tobacco use) and its correlates in Botswana, using a nationally representative survey data. Findings indicate that men, people with no education and primary level education and alcohol users were more likely to report current tobacco smoking. On the other hand, the odds of smokeless tobacco use were significantly higher among women; individuals aged 50-59 and 60-69 years, and individuals with no education and primary education. Findings of this study indicate the need to strengthen existing national policies to reduce harmful use of tobacco among men, women, older adults, no or primary education level individuals and alcohol users.

## Data Availability

The data used for this study is not publicly available due to ethical restrictions but interested parties may contact the authors for more information.

## Abbreviations

AOR: Adjusted Odd Ratios
CI: Confidence Interval
DHS: Demographic Health Survey
LMICs: Low and Middle Income Countries
NCDs: Non Communicable Diseases
SSA: Sub-Saharan Africa
WHO: World Health Organization
